# Effectiveness of External Precooling and Vibration Induced by BUZZY on Pain and Anxiety During Inferior Alveolar Nerve Block Injection in Children

**DOI:** 10.1155/2024/5515522

**Published:** 2024-09-05

**Authors:** Reyhane Narimany, Reyhaneh Faghihian, Mehdi Jafarzadeh Samani

**Affiliations:** ^1^ School of Dentistry Isfahan University of Medical Sciences, Isfahan, Iran; ^2^ Dental Research Center Department of Pediatric Dentistry Dental Research Institute Isfahan University of Medical Sciences, Isfahan, Iran

**Keywords:** behavior management, dental anxiety, external vibration, pediatric dentistry, precooling

## Abstract

**Purpose:** Children's fear of the injection of local anesthetic agents affects their cooperation in pediatric dentistry. Different techniques are available to decrease the injection pain, including the use of precooling agents or vibrators. The present study investigated the effectiveness of Buzzy (Buzzy MMJ Labs, Atlanta, GA, USA). This device transfers cold and external vibration to the injection site during the inferior alveolar nerve block (IANB) injection.

**Materials and Methods:** The present self-control, randomized, and double-blind clinical trial evaluated 30 children aged 6–12, who had bilateral mandibular permanent or primary carious molar teeth. On one side, the BUZZY was applied before and during the IANB injection, and the other side was considered as control. On both sides, a topical anesthetic gel was applied before injection. The pain severity and children's anxiety were determined using Wong–Baker, face, leg, activity, cry, consolability (FLACC) scales, and the heart rate.

**Results:** The mean age of the participants was 7.18 ± 1.5 years, with 12 girls and 18 boys. The Wong–Baker scale and FLACC scale did not show any statistically significant difference between BUZZY and control (*p* value = 0.9 and 0.15, respectively). In addition, BUZZY tool did not significantly decrease pain and anxiety during injection, assessed through the heart rate difference (*p*=0.38).

**Conclusion:** Under the limitations of the present study, a combination of precooling and vibration using the BUZZY device did not decrease pain and anxiety in children during the IANB injection.

## 1. Introduction

Although dental injection is a painful procedure, injecting a local anesthetic agent is inevitable to render pain-free treatment, achieve children's cooperation and comfort, and provide more favorable treatment [1]. Fear of the injection of local anesthetic agents is the most important cause of children's fear in dentistry, resulting in hyperalgesia in children. In addition, a painful experience might increase children's fears in future visits, resulting in a vicious circle. Successful pain control gives rise to cooperation during the treatment sessions, a positive relationship between the dentist and the patient, a higher level of trust, and decreased fear and anxiety in the child. Therefore, dentists have always attempted to find new ways for behavior management in children to control their pain and stress before and during treatment [2–6].

Pain is a subjective and unpleasant feeling produced in response to a stimulus causing tissue injury. Different pharmacologic and nonpharmacologic techniques are available to control pain and manage behavior in children in two categories of pharmaceutical and nonpharmaceutical. Behavior management involves using medications, such as nitrous oxide/oxygen, sedatives, and general anesthetic agents. Nonpharmacological techniques include tell-show-do, voice control, distraction (e.g., through making bubbles or using special distracting cards), and the presence/absence of parents [7]. Other techniques include the application of pressure at the injection site, vibration, precooling of the injection site, heating the anesthetic agent, and surface anesthesia to decrease pain [8–10].

Pain is transmitted to the central nervous system by A-delta and C fibers [8, 11]. There is a mechanism in the spinal cord called gates control, which is responsible for pain control. The sense of vibration is transmitted by A-beta fibers, which block the A-delta and C-fibers that transmit pain; therefore, the patient feels less pain. In addition, vibration accelerates the entry of the anesthetic agent into the bloodstream, decreasing the swelling resulting from it [8, 12]. Several studies have evaluated the efficacy of vibration in decreasing pain resulting from the injections of the anesthetic agent in dentistry, reporting contradictory results [8, 12–17].

Precooling the injection site before injection is an effective, easy, and inexpensive way and has been used for a long time to decrease pain and inflammation in the body. Cold blocks A-delta fibers; therefore, pain impulses are transmitted by C fibers at a lower speed [12].

Different devices have been used to evaluate the efficacy of precooling and vibration in decreasing pain of injection, including Vibraject, Dental Vibe, and BUZZY [3, 6, 13, 14, 18]. The efficacy of BUZZY has been reported at a high level in injecting vaccines and venipuncture and is currently under study in the dental field [9, 18]. It was concluded from a systematic review (2020) on the best intervention to increase the acceptance of the injection of local anesthetic agents by children that further studies were necessary to provide a proper answer to this question, and lack of consensus on the best techniques was reiterated [19]. In another study published in 2022 on the efficacy of Buzzy device, it was shown that this device can be effective in decreasing the pain of infiltration injection in children undergoing dental treatment [20]. Limited studies are available on the use of BUZZY in dentistry. A recently published article assessed the effectiveness of Buzzy on the reduction of pain and anxiety in 3–12-year-old age children [21]. This study showed that Buzzy significantly reduces pain perception during local anesthetic deposition (through infiltration and inferior alveolar nerve block [IANB] techniques) in pediatric patients. The mentioned study is the only one that assessed Buzzy for IANB injection. The results of the mentioned study were contrary to some other studies that can be attributed to different design and pain/anxiety measurement protocols [22, 23]. Two systematic reviews on the efficacy of precooling and vibration showed that BUZZY might provide promising results [15, 24]. In addition, these studies have either had a parallel control design or have evaluated the infiltration technique in the maxilla [22, 25–27]. Since dental fears are related to psychological and physiologic factors in each individual, a split-mouth study is suggested to decrease bias [15, 19].

Considering the quantitative and qualitative limitations of the available studies on the use of BUZZY, and due to the importance of managing pain and stress in pediatric dentistry, the present split-mouth study was designed to evaluate the effectiveness of Buzzy device on pain and anxiety in children during IANB.

## 2. Materials and Methods

The present self-control, single-blind, and randomized clinical trial were carried out in a specialty dental clinic in Isfahan city from October 2020 to June 2021. This study followed the Declaration of Helsinki on medical protocol and ethics. The regional Ethics Review Board of Isfahan University of Medical Sciences approved the study protocol. The study protocol was approved by the Ethics Committee of Isfahan University of Medical Sciences under the code IR.MUI.RESEARCH.REC.1400.115. Informed consent was obtained from parents. In addition, the child was also questioned about his/her interest in taking part in the study.

Thirty children, 6–12 years of age, were included in this study. The inclusion criteria consisted of children with bilateral mandibular carious primary or permanent molar teeth requiring a deep restoration or pulpotomy. The children were treated in two sessions, and their behavior was positive and definitely positive in terms of the Frankl's Behavior Rating Scale (FBRS) [28]. The participants were healthy physically and mentally, had no background medical condition, and were not taking any analgesics or tranquilizers at the time of referral. Children with chronic conditions (asthma, allergy, diabetes, sickle cell anemia, cystic fibrosis, and dermatitis), children with behavioral problems (autism, attention deficit hyperactivity disorder, and learning disorders), children with mental problems, and children with congenital hearing and speech disorders were excluded from the study. If the site planned to receive BUZZY had a pathologic problem or had inflammation, the child was excluded from the study.

In the present study, the participants and the analyzing assistants were blinded. In the first session, after examinations and making the child familiar with the study tools, informed consent was obtained from the parents. Then, the age and FBRS classification were recorded on the checklist. After that, an envelope was used to randomly determine the use of BUZZY in association with the topical anesthetic agent in one of the sessions and only the topical anesthetic gel in the other session. The type of intervention was written on paper and placed in two envelopes, and the child was asked to select one envelope. The selected envelope was considered for the intervention in the first session. Two-week interval was considered as washout period between the treatment sessions. In the intervention session, a nurse placed the BUZZY tool on the skin of the injection site at the angle of the mandible for 2 min. Then, the dentist placed 20% benzocaine surface anesthetic gel (Dentonics, North Carolina, USA) on the injection site using a swab and carried out the IANB injection after 30 s in 2 min. The BUZZY tool was in place during the application of the anesthetic gel and the injection. In the control session, only topical anesthetic gel was applied. To blind the child, the BUZZY tool was placed on the child's face without turning on its vibrator and with its wings at room temperature. After applying the topical anesthetic gel, the IANB was injected in 1 min. A pulse oximeter was used to determine children's anxiety levels based on the heart rate, which directly correlates with the level of anxiety [22]. To this end, the pulse oximeter was connected to each child's index finger on the left side 5 min before the procedure, and the heart rate was recorded. The heart rate was also recorded during the injection and 5 min after it. The Wong–Baker and face, leg, activity, cry, consolability (FLACC) subjective and objective scales were used to determine children's pain severity during the injection. The most reliable method to determine pain severity in children is the self-report and the Wong–Baker Scale is more acceptable than other self-report scales according to children's, parents', and nurses' opinions [14, 29]. The validity and reliability of this scale have been confirmed in the 3−18-year age group [29, 30]. In the Wong–Baker scale, six different faces have been drawn with a score of 10, in which a score of 0 indicates injection without pain and 10 indicates the highest conceivable pain. Immediately after injection, each child was asked to point to the face that accurately described their pain and discomfort level. The FLACC scale is calculated by an analyzer objective scale. In addition, under stressful conditions, this scale can even be more reliable than the self-report. In this scale, the child's body status, including the face and feet and activities, crying, and adaptation, are scored from 0 to 2 according to a table and summed up to achieve a score from 0 to 10. If the score is close to zero, the child has experienced no pain. If the score is 1–3, the child has experienced mild pain and discomfort. If the score is 4–6, the child has experienced moderate pain, and if the score is 7–10, the child has experienced severe pain. FLACC behavioral pain scale was previously found to have excellent validity and reliability for pain assessment in young, cognitively intact children [30].

To determine this scale and ensure blinding, a camera was placed out of the child's visual field to always record films from one angle. The film was viewed by an operator not involved in the study process, who was unaware of the study procedures and process. In the first stage, the film was muted, and the observer scored the first three statuses (i.e., the face, feet, and activity). In the second stage, the film's sound was played, and the remaining statuses (crying and adaptation) were scored. The data were analyzed with Chi-square, ANOVA, and *t*-test.

## 3. Results

**Figure 1:**
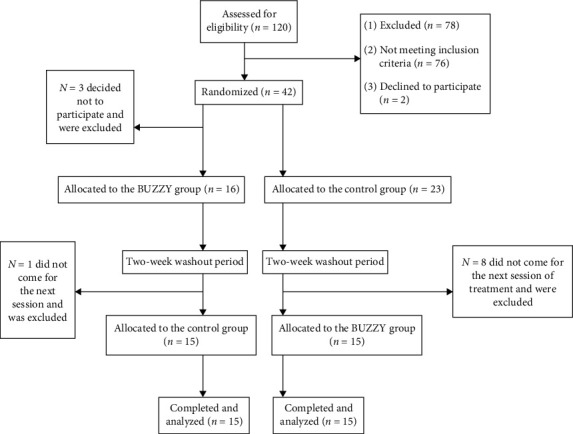
Flowchart of the participants of clinical trial.

The present study evaluated the effectiveness of precooling and external vibration on the severity of pain and anxiety during IANB injection in children 6–12 years of age, with a mean age of 7.18 ± 1.50. Forty-two children were included in the study, but 12 were excluded because they did not return for the second visit. Finally, 30 children completed the study, that is, 60 IANB injections were carried out. Fifteen children received the intervention IANB + BUZZY in the first session (Group A), and 15 children received the intervention IANB + BUZZY in the second session (Group B).  Figure 1 shows the flowchart related to patients participated in this study as well as reasons for exclusion (Figure 1). Twelve children (40%) were female, and 18 (60%) were male, with no significant difference in gender between the two groups (*p*=0.456).

Table 1 presents the means and standard deviations of the Wong–Baker, FLACC, and Diff_PR (pulse rate difference before and after the intervention; PR1 – PR0) in the test and control sessions in Groups A and B. Intragroup analysis of mean Wong–Baker, FLACC, and Diff-PR scores of Groups A and B (treatment effect) did not show significant differences between intervention and control sessions in each group (*p* value = 0.9, 0.15, and 0.38, respectively). Additionally, intergroup analysis of mean FLACC, and Diff-PR scores of first and second sessions of Groups A and B (period effect) did not show significant differences (*p* value = 0.97, 0.51, respectively). However, the difference was significance for Wong–Baker score (*p* value = 0.04). Moreover, application of buzzy in the first versus second session (carry out effect) did not change the Wong–Baker, and Diff-PR scores significantly (*p* value = 0.09, 0.09, respectively). In contrast, significant difference was found in FLACC scores (*p* value = 0.03).

Table 2 compares the parameters in the whole study population (*n* = 30) without considering priority of using BUZZY. There are no significant differences in none of the Wong–Baker, FLACC, and Diff-PR score (*p* value = 0.80, 0.56, and 0.46, respectively).

## 4. Discussion

Successful treatment in dentistry is not possible without injecting local anesthetic agents. Most cases of fear and anxiety of patients in dentistry are related to the injection of the anesthetic agents [4, 31]. Skilled dentists should be able to render proper treatment and foster trust and positive attitudes in their patients at the same time. To achieve this aim, the dentist should implement appropriate behavior management strategies, the prerequisite of which is pain control [8]. Different techniques are available for pain control. In the present study, a combination of precooling and external vibration with a BUZZY tool was used. The results of the present split-mouth trial showed that the BUZZY tool did not decrease children's pain and anxiety during the IANB injection. Several studies have shown that tools creating vibration can decrease children's pain and anxiety using the distraction technique [25, 26, 32]. However, the present study did not confirm this. In the studies mentioned above, the children did not have a preliminary session to become familiar with the study tool; therefore, they might have been distracted in the treatment session.

Pain is affected by different factors, including anxiety, fear, previous experience, personality, trust in the dentist, and a feeling of control over the situation; therefore, it is difficult to determine pain severity [33, 34]. The necessity of carrying out a split-mouth study to survey pain and anxiety becomes clearer when individual differences are considered. Different individuals have different pain thresholds, psychological characteristics, and gender-related differences [15]. One of the advantages of the present study over previous studies is its cross-control design [22, 26, 32].

Pain severity is challenging in children due to limited verbal communication. Therefore, it is advisable to combine the three parameters for pain survey, including self-report, behavior assessment, and physiologic responses in children [34, 35]. The gold standard for pain assessment in children is the self-report method, and of all the available self-report methods, children prefer to show their pain levels using pictures [29, 36]. Therefore, the Wong–Baker scale was used in the present study, in which the mean pain scores in the precooling and vibration session in both Groups A and B were less than that in the control session. However, statistically significant difference was not seen. In medical procedures, such as venipuncture, some studies have been carried out [9, 35], Potts et al. [35] studied the use of the BUZZY during the venipuncture in 4–18-year-old patients in a parallel design study, concluding that the Wong–Baker scale scores were not significantly different between the two groups. Studies have shown that children with anxiety, even if they do not experience any pain, are disposed to show the uncomfortable face as an indication of their pain. One of the disadvantages of scales with facial pictures is that they show pain severity higher than what it really is [32, 37], which might explain the lack of difference between the two groups. The results of the present study are contrary to a study by Bilsin, Güngörmüş, and Güngörmüş [26], which might be attributed to the split-mouth nature of studies compared to parallel studies. Alanazi, Pani, and AlGhanim [25] carried out a split-mouth study and showed the efficacy of the BUZZY technique in decreasing pain severity in 7-year-old children during the infiltration technique. The IANB injection which was used in the present study is a more painful technique for children and a challenging injection for dentists than the infiltration technique, and the difference in ithe result can be attributed to the difference in techniques used [38, 39].

Observing their behavior might be a reliable method to assess very young children's pain under difficult and stressful conditions [31]. To this end, the FLACC scale was used in the present study. The mean FLACC score in the present study in children receiving the BUZZY method in the first session was slightly less than in the control group; in children receiving BUZZY in the second session, these scores were slightly higher than those in participated as control group in the second session. Finally, based on the FLACC scores from a statistical viewpoint, BUZZY did not result in significant decreases in pain severity. The results of the present study are consistent with a study by Potts et al. [35], who used the FLACC index in addition to the Wong index during venipuncture. The results of the present study are different from those of a study by Alanazi, Pani, and AlGhanim [25], who evaluated the efficacy of BUZZY during the maxillary infiltration injection in the maxilla, concluding that BUZZY was significantly effective in decreasing pain. Such a discrepancy in the results of studies might be attributed to the type of injection. AlHareky et al. [27] conducted a parallel arm study using BUZZY in maxillary infiltration. In the FLACC index, there was a significant decrease in pain severity in the BUZZY group. However, the sound, eye, and motor (SEM) index, which is a behavioral index, did not reveal a significant difference between the intervention and control groups [27]. This study had a parallel control design, which has a higher rate of bias than cross-sectional control studies [15]. In addition, they did not exclude the children who had a history of dental treatment.

According to the results, in the first treatment session, BUZZY was more successful than the anesthetic gel in decreasing pain severity. Since children are more fearful in the first session, it is highly important to manage pain in the first session [8]. Further studies are suggested in this respect.

In the present study, the heart rate was selected as a physiologic scale to assess pain and assess pain and anxiety. Increased heart rate indicates pain and discomfort. In addition, an increase in anxiety level increases the heart rate [25, 40]. In the present study, the mean difference in heart rates before and during injection did not significantly decrease in any of the sessions, neither in Group A nor in Group B. However, two previous parameters decreased significantly when BUZZY was applied, leading to the conclusion that children had a better attitude toward anesthesia in the first session while BUZZY was applied than when the anesthetic gel was used alone; however, children's anxiety did not decrease. Chaudhry et al. [17] carried out a split-mouth study to evaluate the effect of internal vibration by Vibraject during the alveolar nerve block injection, reporting no significant difference in heart rates between the intervention and control sessions. Suohu et al. [22] carried out a parallel-control study and reported no significant differences in heart rates between two groups of subjects. The results of these studies and the present study are contrary to a study by Sahithi et al. [33] in a parallel-control study. In the study mentioned, the subjects were 4−11-year-old children who were positive and negative on the FBRS scale; however, 6−12-year-old children who were positive and definitely positive according to the FBRS scale were included in the present study. In addition, the control group in the study above had undergone counterstimulates, and a local anesthetic gel was not used. Dental anxiety is related to the individual's psychological and physiologic factors. Some of the reasons for children's anxiety are children's cognitive abilities, parents' anxiety, and past experience [19]. In the present study, children with a past dental experience were excluded; however, the two other variables should have also been limited to achieve more accurate results.

The present study had some limitations. The results of the present study can only concern cooperative children and those receiving the IANB injections, and they cannot be extended to noncooperative children who need other injections and are <6 years of age. The operator recording heart rates and questioning the children about their pain were not blinded. It is suggested that in future studies, the efficacy of BUZZY be evaluated in the 3.5−6-year age group. In addition, the children should be asked to score the Wong–Baker scale before injection, too. Other scales, such as SEM, a behavioral scale, and children's fear scale, a self-report scale for evaluating pain, can be used.

## 5. Conclusion

Promising results were achieved concerning the use of BUZZY in the first session to decrease children's pain. However, further studies are necessary.

The following are the limitations of the present study:

i. The BUZZY tool did not significantly decrease dental pain in 6−12-year-old children during the IANB injection.
ii. The BUZZY tool did not decrease the dental anxiety of children.

## Figures and Tables

**Table 1:** The means and standard deviations of Wong–Baker, FLACC, and DIF_PR in Groups A and B (ANOVA).

**Scales**	**Groups**	**Group A** **(mean ± SD)**	**Group B** **(mean ± SD)**	**Treatment** **effect** **(*p* value)**	**Period effect** **(*p* value)**	**Carry out** **effect** **(*p* value)**
WB	Buzzy	2.66 ± 3.43	6.93 ± 3.99	0.9	0.04	0.09
	Control	5.2 ± 4.32	3.86 ± 3.66			

FLACC	Buzzy	1.53 ± 2.38	2.6 ± 1.95	0.15	0.97	0.03
	Control	3.13 ± 2.29	1.66 ± 2.02			

DIF_PR	Buzzy	3.93 ± 13.61	14 ± 13.47	0.38	0.51	0.09
	Control	8.93 ± 15.54	3.4 ± 8.67			

Abbreviation: DIF_PR = PR before injection − PR during injection.

**Table 2:** The means and standard deviations of Wong–Baker, FLACC, and DIF_PR of all participants in the intervention and control groups (participants in both A and B groups) (*t*-test).

**Groups**	**Treatment**	**Total number of participants who received treatment in both groups**	**Mean ± SD**	***p***
WB	Buzzy	30	4.8 ± 4.25	0.80
	Control	30	4.5 ± 3.99	

FLACC	Buzzy	30	2.06 ± 2.21	0.56
	Control	30	2.4 ± 2.25	

DIF_PR	Buzzy	29	8.79 ± 14.25	0.46
	Control	30	6.17 ± 12.69	

## Data Availability

Data of this research are available upon request.
